# Barriers and facilitators to employment in borderline personality disorder: A qualitative study among patients, mental health practitioners and insurance physicians

**DOI:** 10.1371/journal.pone.0220233

**Published:** 2019-07-23

**Authors:** Trees T. Juurlink, Miljana Vukadin, Barbara Stringer, Marjan J. Westerman, Femke Lamers, Johannes R. Anema, Aartjan T. F. Beekman, Hein J. F. van Marle

**Affiliations:** 1 Department of Psychiatry, Division of GGZ inGeest Specialized Mental Health Care, Amsterdam UMC, Vrije Universiteit, Amsterdam, The Netherlands; 2 Department of Social Medicine, Amsterdam UMC, Vrije Universiteit Amsterdam, Amsterdam, The Netherlands; 3 Centre for Consultation and Expertise (CCE), Utrecht, The Netherlands; 4 Department of Health Sciences, Amsterdam UMC, Vrije Universiteit Amsterdam, Amsterdam, The Netherlands; CentERdata, NETHERLANDS

## Abstract

**Background:**

Borderline personality disorder (BPD) is associated with unemployment and impaired functioning. However, a comprehensive understanding of barriers and facilitators to employment from a multidisciplinary perspective is currently lacking. Therefore, the aim of this qualitative study was to explore barriers and facilitators in gaining and maintaining employment in BPD from the perspectives of patients, mental health practitioners (MHPs) and insurance physicians (IPs).

**Methods:**

Fifteen semi-structured interviews were conducted in patients with BPD and two focus groups were carried out among MHPs (n = 7) and IPs (n = 6) following a thematic content analysis approach.

**Results:**

All participants described barriers and facilitators relating to three overall themes: characteristics of BPD, stigma, and support to employment. Barriers to employment mainly related to characteristics of BPD, such as low self-image, difficulty posing personal boundaries, difficulty regulating emotions, and lack of structure. MHPs and IPs additionally mentioned externalization and overestimation of competencies on the part of patients. Enhancing emotion regulation and self-reflection by successful treatment was suggested as a facilitator to enhance employment. Increasing collaboration between mental health and vocational rehabilitation services, and increasing knowledge about BPD, were suggested to increase sustainable employment and decrease stigma.

**Conclusions:**

The present findings revealed that both facilitators and barriers are important in gaining and maintaining employment in BPD in which diminishing symptoms, examining stigma and increasing support to employment are key. As a next step, supported employment strategies that follow patient preferences and integrate employment and mental health services, should be studied in the context of BPD.

## Introduction

Borderline personality disorder (BPD) is a severe mental disorder characterized by an enduring and pervasive pattern of instability of interpersonal relationships, self-image and affects, marked by impulsivity and (para)suicidal behaviors [1]. In western societies, prevalence estimates range from 1 to 1.5% in the general population [2–4] to 10 to 20% in clinical populations [4–6]. Unemployment and difficulties in gaining and maintaining employment are highly prevalent in BPD and add to social exclusion, and deterioration of physical and mental health [7–12]. Individuals with BPD however, express a strong wish to gain employment as working contributes to feelings of competence and being ‘normal’ [13]. From a societal point of view, the high costs concerning occupational disability of individuals with BPD provide further reasons to improve employment within this group [14–20].

In general, unemployment and disability benefits are common in individuals with mental health disorders [21]. Barriers to employment from the perspective of individuals with mental health disorders are stress, stigma, fear of loss of benefits, low expectations, and lack of follow-up support [22–24]. Lack of collaboration between mental health and vocational rehabilitation services also hampered return to employment [25–28]. Furthermore, stigma impedes employment in three ways: (i) fear of disclosure, (ii) negative attitude of employers, and (iii) anticipated stigma [29]. Facilitators to employment involve having a work history, and professional support during job search and during employment [23,30–33].

With respect to employment in BPD, a review study has showed that roughly 50% of individuals with BPD manage to find employment [34]. However, only 20% of those in employment are capable of maintaining employment and becoming financially independent of social benefits. Jovev & Jackson [8] explain these low rates by showing that BPD patients experience high levels of stress and malfunctioning during work. Furthermore, Sio and colleagues [35] showed that impulsivity in individuals with BPD was associated with poor employment outcomes after 12 months. Moreover, BPD is characterized by a pattern of instability in interpersonal relationships, disturbed self-image and affect, and impulsivity [1,6], which conceivably all result in impaired functioning in employment settings. Another potential barrier to employment that is significant in BPD is stigma [36]. Specifically, stigma from mental health care professionals towards BPD is a well-known problem [37–40]. There is currently no literature yet on stigma towards BPD from insurance physicians (IPs). In the Netherlands, IPs are mandated to judge the medicolegal eligibility of claims for a sickness and work disability benefit supplied by the Dutch Social Security Agency (SSA) and provide sociomedical guidance to sickness benefit claimants to return to work. It is known, however, that knowledge-related and attitude-related barriers were found to impede IPs guideline adherence in mental health [41].

So far, research on gaining and maintaining employment in BPD is scarce, especially research that combines a multidisciplinary perspective involved in the pathway to work, such as from mental health practitioners and insurance physicians. Furthermore, as of yet, the described barriers to employment in BPD do not directly provide strategies to improve practice. Therefore, the main objective of this qualitative study is to explore the barriers and facilitators of gaining and maintaining employment in BPD in patients, mental health practitioners (MHPs) and insurance physicians (IPs). Qualitatively exploring these factors provides the opportunity to reveal unexpected themes. Subsequently, these factors will be examined in order to assess the needs for vocational rehabilitation strategies (like Individual Placement and Support, IPS) and ultimately increase employment rates in individuals with BPD.

## Methods

### Design

A qualitative explorative study using semi-structured interviews in patients and focus groups in MHPs and IPs was performed to collect rich and in-depth data on barriers and facilitators to employment in BPD.

### Context

In the Netherlands mental health and vocational rehabilitation are separate services. Although the current dominating vocational rehabilitation method for patients with severe mental illness is IPS, other patient groups typically receive stepwise vocational trajectories, putting more emphasis on assessments of individual competencies and connecting prevocational activities [42].

Most BPD patients that receive (psychotherapeutic) treatment are treated in outpatient clinics. Additionally, patients with BPD can be treated in the multidisciplinary setting of acute mental health (aimed at short-term care instead of cure) or so-called Flexible Assertive Community Treatment (FACT) (providing extensive care through a combination of individual case management and home visits) [43].

### Sample and data collection procedures

#### Patients with BPD

Patients were recruited from an outpatient clinic for personality disorders of a mental health care institution in an urban area of the Netherlands, serving over 200 patients. In order to be eligible for participation, individuals had to be primarily diagnosed with BPD and fluent in Dutch. Participants were invited by an invitation letter from their practitioner explaining the aims of the study. If individuals met inclusion criteria and were willing to participate, they were contacted by the researcher (TJ). The researcher explained the objectives of the study and scheduled an interview. Between March and July 2017, 16 individuals agreed to participate in the study. Interviews were conducted at a time and location convenient for the participants and generally took place at the outpatient clinic within three weeks following participant inclusion. Before the start of the interview, written informed consent was obtained. In this consent, participants also authorized the authors to use clinical characteristics from the DSM diagnoses, predominantly based on SCID interviews. The recruitment of new participants stopped when no new themes emerged from the interviews [44]. After approximately 12 interviews no new themes occurred, three more interviews were conducted to ensure saturation. One interview could not be scheduled within the timeframe of data collection, resulting in a total sample size of 15 semi-structured interviews in patients with BPD.

The topic list was designed with the research group using topics from previous studies in employment and mental health in general [32,45,46]. The following topics were discussed: experiences with employment, barriers and facilitators to employment, stigma and disclosure of BPD (see S1 File). The interviews were held with this topic list. During the interviews with patients and in both focus groups we consistently aimed to distinguish the barriers and facilitators originating from BPD from those originating from possible comorbid disorders. The interviews were held by the first author (TJ), female, trained in qualitative research methods. All interviews were audiotaped and transcribed verbatim. The interviews lasted on average 1 hour (range 30–105 minutes). Field notes and memos were made for analyzing purposes during and after the interviews. For this manuscript, a native English speaker translated the citations from Dutch.

#### Mental health care professionals and insurance physicians

To be eligible, both professional groups had to have experience in working with patients with BPD for at least 6 months. For the focus group with MHPs, one member of the research team (HvM), psychiatrist, informed and invited other practitioners from the outpatient clinic. The invitation for participation in the focus group was initially send out to all practitioners working at the outpatient clinic for the specialized treatment of patients with personality disorders consisting of 63 individuals. After obtaining a low response rate, 25 practitioners were approached by email again, but 18 declined due to conflicting appointments or holidays. Seven MHPs were willing to participate in the focus group interview at May 18^th^ 2017 lasting 100 minutes. However, MHPs (and IPs) were asked about their experiences with patients with BPD in general (and thus data was not analyzed as specific patient-professional dyads).

For the focus group with IPs, a member of the research team (JA), insurance physician, invited twelve IPs from a bimonthly meeting at the SSA. Half of the group declined due to conflicting appointments or maternity leave, however six IPs were able to participate in the focus group interview on June 8^th^ 2017 lasting 95 minutes. Participants were all employed at the SSA, working at different offices in urban areas in the Netherlands. IPs were asked to share their experiences with patients that had a recorded BPD diagnosis by a qualified mental health professional.

At the start of the focus group each participant was asked to write down one word they associated with employment in individuals with BPD on a memo to provoke conversation about (different) perspectives. The memos were pasted on a whiteboard and each participant was invited to explain their word. Furthermore, each theme from the topic list was introduced with a statement. The discussion allowed for further exploration of how the different barriers and facilitators interacted. Subsequently, participants were invited to share possible solutions to improve employment in BPD. Both focus group interviews were moderated by BS and assisted by TJ and held at the workplace of the participants. All interviews were audiotaped and transcribed verbatim. Field notes and memos were made analyzing purposes during and after the interviews.

#### Analysis

A thematic content analysis approach was used [44]. The transcripts were summarized by the first author (TJ), and provided to all participants for member checking [44]; no requests for changes occurred. Atlas.ti software (version 6) was used to facilitate data management and analysis. TJ thoroughly started reading all transcripts. The analyses started with independent coding of five information rich transcripts by TJ and MV. From this, a preliminary codebook was established by TJ and MV based on consensus by discussion. Two semi-structured interviews, the summaries of the focus groups, and the codebook were discussed with BS and MW. The data was studied case-by-case by reading and re-reading the transcripts, memos and field notes and discussing the codes and themes derived up until agreement. By analyzing the data in comparison to the other transcripts, codes were sorted and merged, and themes were created together with MV and BS. The themes were reviewed, focusing on understanding the collected data and reassuring that the data still corresponded to the themes assigned. Finally, the findings were critically discussed with all authors.

#### Ethical considerations

The science committee of GGZ inGeest (CWO) approved the study and the Medical Ethics Committee of the VU University Medical Center (METC) declared that the study does not fall within the scope of the Medical Research Involving Human Subject Act (2017.092). All procedures performed in this study were in accordance with the ethical standards of this institutional research committee and following the principles of the Helsinki declaration. Written informed consent was provided by all patients with BPD.

## Results

Participating patients with BPD represented a heterogeneous group with respect to employment, varying from recent or long-term employment or unemployment to having multiple jobs in their employment history, see Table 1. The type of employment was also diverse (S1 Table).

**Table 1:** Socio-demographic and clinical characteristics of patients with BPD.

	**Patients with BPD (n = 15)** **N (%)**
Gender	
Female	14 (93)
Age	
Mean (range)	39 (23–58)
Employment	
Employed	4 (27)
Unemployed	11 (73)
with voluntary job, internship or unregistered job	6 (55)
Partnership	
Living alone	8 (53)
Living with partner/family	7 (47)
Co-morbid diagnoses	
Any other PD	2 (14)
Depressive disorder	4 (27)
Substance use disorder	4 (27)
PTSD	2 (14)
Generalized anxiety disorder	2 (27)
Eating disorder	2 (14)
Bipolar disorder	1 (7)
Dissociative disorder	1 (7)
AD/HD	1 (7)

PD: Personality disorder

PTSD: Post-Traumatic Stress Disorder

AD/HD: Attention Deficit Hyperactivity Disorder

Participating professionals differed in age, years of experience in working with patients with BPD, discipline (in the MHP group), and sex (primarily in the IP group), see Table 2.

**Table 2:** Characteristics of mental health practitioners and insurance physicians.

Mental health practitioners (MHPs) (n = 7)*	
Age	
Mean (range)	50 (31–65)
Sex (n)	
Female	6
Position	
Psychiatrist	1
Psychologist	3
Behavioral therapist	2
Occupational therapist	1
Number of years of experience working with BPD	
Mean (range)	12.9 (1–30)
**Insurance Physicians (IPs) (n = 6)**	
Age	
Mean (range)	51.5 (41–64)
Sex (n)	
Female	3
Number of years of experience working with BPD	
Mean (range)	18.7 (10–30)

BPD: Borderline personality disorder

* All participants worked in different teams (including specialist services in dialectical behavioral therapy, mentalization-based treatment and schema-focused therapy), of the same outpatient clinic for the specialized treatment of personality disorders, from which the patients were also recruited.

Several themes emerged from the data as barriers and facilitators to employment. The overarching themes were classified into: characteristics of BPD, stigma and support to employment. Most barriers and facilitators were interchangeably connected as the identified barriers and facilitators related to similar features, see Table 3. No participants were currently enrolled in a vocational rehabilitation program, however few participants had previously received general vocational rehabilitation services. Nonetheless, no participant had experience with IPS. Support to employment in the following text refers to all previous vocational rehabilitation services provided to patients in this study.

**Table 3:** Barriers and facilitators to employment in BPD from the perspectives of patients, MHPs and IPs.

**A. Characteristics of BPD**		**Patients**	**MHPs**	**IPs**
Barriers	Low self-image	*	*	
	Fear of making mistakes	*	*	
	Previous experiences of failure → increase low self-image	*	*	
	Rumination	*		
	Mood swings	*	*	*
	Difficulty posing personal boundaries	*	*	*
	Feeling responsible	*	*	
	Impulsive behavior	*	*	*
	Difficulty in regulating emotions	*	*	*
	Lack of structure/overview	*	*	*
	Externalization		*	*
	Overestimation		*	*
Facilitating characteristics in patients with BPD	Ambitious	*	*	*
	Hardworking	*	*	
	Entrepreneurial	*	*	*
Proposed facilitators to target impeding characteristics	Amplifying self-reflection and regulation of emotions		*	*
	Treatment (to improve regulation of emotion, self-image, sensing and posing personal boundaries and structure and overview)	*	*	
**B. Stigma**		**Patients**	**MHPs**	**IPs**
Barriers	Discouragement of disclosure and/or fear of disclosure of BPD	*	*	*
	Stigma in BPD	*	*	*
Proposed facilitators	Renaming BPD into emotion regulation disorder	*	*	
	Relabeling of BPD by positively campaigning BPD		*	
	Development of a ‘manual’ that describes symptoms and how to cope with these symptoms and encourage disclosure		*	*
**C. Support to employment**		**Patients**	**MHPs**	**IPs**
Barriers	Lack of support	*	*	*
	Misconception (about BPD) in vocational rehabilitation	*	*	
Proposed facilitators to improve (support to) employment	Increase collaboration between services	*	*	*
	Integrate vocational rehabilitation services within treatment regimen	*	*	
	Increase knowledge of BPD and treatment perspectives to align treatment with vocational rehabilitation			*

*: Identified in subgroup

BPD: Borderline personality disorder

MHPs: Mental health practitioners

IPs: Insurance physicians

### A. Characteristics of BPD

#### Barriers according to patients with BPD

All patients related their problems with gaining and maintaining employment primarily to symptoms of BPD. How patients coped with their symptoms in relation to employment varied widely. Overall, they described having a low self-image that hindered employment for instance through a fear of making mistakes as exemplified by participant 14: *“Well*, *being insecure with respect to my job*, *not knowing whether I performed up to standard*. *[*..*] For six years I had great difficulty keeping up my work and meeting expectations*, *so that they didn’t think I was weird or something*. *That made me feel lonely and most of all it wasn’t clear to me what they expected from me”*. This (further) decreased self-image and resulted in a ‘downward spiral’ of negative thoughts, as exemplified by participant 13: *“It feels as if I am the stupidest person in the world*, *I feel worthless and then I end up in a downward spiral*. *I remember all the previous mistakes I made until I come to a point where–when it’s really bad–I’ll think ‘Well*, *I’ll just cut my wrists now’”*. Patients with BPD noted having high expectations of themselves while simultaneously failing these expectations and ruminating about how others might perceive them. Also, rapid mood swings caused problems to comply with previously made appointments, mostly due to instantly and unpredictably feeling depressed or behaving impulsively.

Strong feelings of responsibility led to taking up too much work, as explained by participant 7: *“About communicating my own boundaries*. *I am continuously crossing them myself and find it hard to communicate them to others at my work*. *Often I am too compliant and I end up saying*: *“Ok*, *I’ll do it”*. This ongoing internal process led to exhaustion described as “a ticking time bomb that eventually bursts”. A general difficulty to regulate emotions further complicated things resulting in either impulsive (often conflictual) or avoidant behavior as described by participant 1: *“For too long I will see things I don’t agree with at my job*, *but I don’t dare to say anything about it*. *I just continue working*. *Then eventually I will have an outburst”*.

Most patients mentioned having problems in several domains of life such as social, financial and their living situation. Also, comorbidity with other mental disorders such as affective and substance use disorders was frequent. This, in combination with their feelings and behavior, was described as an interchangeable process of increased loss of structure and overview, also noticeable in work as described by participant 12: *“I kept forgetting the weirdest things*, *for example I kept losing receipts of registered mail as well as things that were send to me*. *I could not understand how I could lose them*. *I thought to myself*: *‘Yes*, *I stored them carefully*.*’ It drove me crazy which aggravated the confusion and made me feel even more stressed*. *And the more stressed I got*, *the more things went wrong*, *still not understanding what was going on”*.

#### Barriers according to mental health practitioners and insurance physicians

Both MHPs and IPs described similar BPD-related characteristics that impeded employment, however they provided different descriptions. MHPs explained how a low self-image was maintained due to being easily offended: *“They [patients with BPD] have a tendency to feel at a disadvantage*. *If*, *for example*, *somebody raises an eyebrow in a certain way*, *a person with BPD can feel attacked*, *not taken seriously and not validated”*. Furthermore, MHPs reasoned how individuals with BPD are often misunderstood: *“It seems that individuals with BPD are good at posing personal boundaries*, *while in fact they are not*. *Often they pose them too late or too little*. *A lack of assertiveness or interpersonal skills really*. *And that causes the tension to rise”*.

According to IPs, mood swings and impulsive behavior in BPD were due to a lack of self-reflection. This contrasts with the descriptions provided by patients and MHPs, they stated that mood swings and impulsive behavior were caused by low self-image. Furthermore, black and white thinking and externalization impeded gaining and maintaining employment according to MHPs and IPs. This is because externalization caused difficulty in evaluating previous (conflictual) situations and mitigated self-awareness in individuals with BPD.

Also overestimation of capacities was mentioned as a problem to employment as stated by an IP: *“In itself patients with BPD are good at ‘selling’ themselves*, *so at least in the beginning you’re impressed*. *However*, *when it comes down to it they perform poorly which tends to irritate employers*. *Realizing a goal is possible*, *but very often not together with colleagues*, *which makes it hard*. *Besides*, *it’s not only the patients that overestimate themselves*, *it’s also their environment”*. Another IP stated: *“And if they [patients with BPD] overestimate themselves it becomes very difficult to find a suitable job*, *because if they like a job*, *you often think it’s not realistic”*. Furthermore, IPs noted that patients with BPD typically pursue jobs that trigger symptoms of BPD, and IPs therefore declared these jobs as unsuitable. For example, patients with BPD often wanted to work with vulnerable people. Eventually, this compassion for others often turned into a barrier due to a lack of posing personal boundaries and becoming overly involved until they call in sick or act impulsively.

#### Proposed facilitating characteristics in relation to barriers according to all participants

The following characteristics in patients with BPD were described as facilitators to employment: working hard, being entrepreneurial, ambitious and passionate, and having various interests. MHPs described that patients with BPD, despite the association with dysfunctional interpersonal skills, are emphatic and sensitive to others. However, all groups came to realize that these facilitating characteristics could easily change into barriers. An MHP noted: *“Often*, *at least at the beginning*, *they [patients with BPD] have a certain energy and enthusiasm that can be contagious for co-workers*. *They feel like they’re starting over with a clean slate and are highly motivated*. *So*, *as long as that period lasts*, *I can imagine that employers are happy with them”*. An individual with BPD exemplified how her drive (as a facilitator) could turn into a barrier, participant 13: *“In retrospect I can think ‘give yourself a break’*, *but at that moment I just have to succeed*. *Somehow*, *I take it all too seriously*, *I want to do well and I run the risk of losing myself in my work*. *If then it doesn’t work out*, *I feel so responsible that I can literally freak out”*.

Patients described how treatment helped them to better understand their feelings by learning to regulate their emotions. Treatment furthermore improved recognizing feelings and corresponding behavior, as summarized by participant 1: *“[Being in treatment] taught me to handle things differently*. *I observed my own behavior and came to realize that I should stop pointing my finger at other people*. *It’s not ‘he*, *he*, *he’ or ‘she*, *she*, *she’*, *it’s ‘me’*. *My psychologist taught me to stop being a victim [*..*] He told me*: *‘it is up to you’ and I knew he was right*, *I just did not know yet how to do things differently”*. MHPs emphasized that treatment is furthermore needed to increase self-image and self-awareness and improve sensing and posing personal boundaries. Treatment also contributed to diminish the stress experienced from problems in various life domains by helping to increase overview and structure. According to all participants diminishing impeding BPD characteristics was necessary before return to employment, however IPs were unfamiliar with treatment prospects in BPD.

### B. Stigma

#### Barriers according to patients with BPD

Some patients with BPD gave examples of being fired due to (involuntary) disclosure of their diagnosis. However, two participants had good experiences with disclosure. All, except these two participants, would not disclose their diagnosis in the future because they believed BPD is being stigmatized. They felt that disclosure would abate their chances to gain employment, or expressed not to know how to disclose their diagnosis in a constructive manner. These patients felt that it would be better to either describe mere BPD symptoms or disclose any other diagnosis because of the stigma surrounding BPD, as exemplified by participant 12: *“I had to fill out a form about mental illness*. *I am open about that*, *although I didn’t use the term borderline*. *Instead I stated that I am suffering from a depression*, *because there is a lot of overlap between the two disorders and I think the term borderline has too much negative connotations”*.

#### Barriers according to mental health practitioners and insurance physicians

MHPs described how the name BPD and the corresponding stigma resulted in anticipated stigma in patients with BPD. More specifically, MHPs explained how the ‘label’ BPD confirmed the low self-image already present in patients with BPD. Simultaneously, during the focus group MHPs realized they were stigmatizing themselves and tended to think that patients with BPD would not recover from their disorder. IPs also noted having little hope about the capabilities of patients with BPD in relation to employment, one IP stated: *“We are stigmatizing them too I guess*. *[*..*] You develop a prejudice based on previous experiences*. *In a way that you think*: *‘this will never work”*. Moreover, both professional groups would not recommend disclosing BPD to potential employers. Simultaneously, they realized that this induces preservation of the stigma surrounding BPD, as exemplified by an MHP: *“But in fact we’re part of the problem of stigmatization [*..*] Apparently we all agree with them that it’s better not to disclose their diagnosis”*.

#### Proposed facilitators to target stigma according to all participants

MHPs specifically mentioned it was essential to ‘relabel’ BPD in order to target stigma. This positive ‘relabeling’ should be done through mental health care and anti-stigma programs. This relabeling should include 1) renaming BPD, for instance in emotion regulation disorder (as preferred by both patients and MHPs), 2) promoting the positive features of patients with BPD in relation to employment in the public (for instance in the form of a campaign as has been previously done for autism spectrum and depressive disorders), and by educating the general public about BPD as exemplified by an MHP: *“Psychoeducation is needed to lessen the stigma surrounding BPD*. *For instance*, *it is important to communicate that there are multiple evidence-based treatments available for BPD”*. Moreover, both MHPs and IPs suggested to develop a ‘manual’ for employers, co-workers and patients with BPD themselves in which both symptoms of BPD are described and how to cope with these symptoms.

### C. Support to employment

#### Barriers in support to employment according to patients with BPD

With regard to reintegration services, some patients with BPD expressed feeling being set aside, as exemplified by participant 9: *“I had the idea that the vocational rehabilitation service from the SSA just stopped calling me*. *Probably because they gave up on me and thought I would not recover”*. Although some IPs allowed patients with BPD to undergo treatment before restarting work, there were also patients who felt pressured by IPs to return to employment as soon as possible regardless of their mental health as exemplified by participant 10: *“They just follow the protocol and try to reach their targets*. *They’re insensitive to your arguments*. *They just wait and see how you respond*. *I think that is the idea because I provided the IP with contact information of my clinicians and my entire treatment history*, *but he just didn’t hear it*. *Up to the point that I became emotional and asked*: *‘Do you get it*?*’*. *And he just replied ‘Yes*, *I know what you’re after’*, *in other words ‘I know that you want to continue receiving sickness benefits’*. *Then I think by myself ‘You really do not take the effort to understand”*.

#### Barriers in support to employment according to mental health practitioners and insurance physicians

MHPs acknowledged there was little attention for employment in most treatment programs. Initiatives for support to employment often came from patients with BPD themselves, that were subsequently referred to the departments’ occupational therapist. There was however, one treatment program that devoted sessions to post-treatment employment.

IPs acknowledged having a lack of knowledge in treatment prospects for BPD. Also, IPs noted that collaboration between mental health and vocational rehabilitation services was lacking. One IP however (from another region), stated that their office had a fruitful collaboration with mental health institutions marked by frequent counselling and educating each other. IPs addressed that it was difficult to assess working capability for someone with BPD because based on the criteria of disability insurance, patients with BPD are mainly assessed as eligible for (certain types of) employment. However, IPs simultaneously realized that in order to increase sustainable employment it might be necessary to reduce BPD symptoms first.

Furthermore, an MHP stated that the SSA treats patients with BPD differently than patients with other mental disorders: *“I have multiple examples of insurance physicians who state that a personality disorder is not a medical condition [and for that reason do not advice sickness or social security benefits]*, *while a depressive disorder is”*.

#### Proposed facilitators necessary to improve (support to) employment in BPD according to all participants

All participants acknowledged the importance of employment. Furthermore, all patients with BPD wanted to be employed and expressed hope in achieving this goal, although two patients realized return to competitive employment was no longer feasible for them (one was found to be incapacitated for work according to the Work Capacity Act (WAO)). Another participant described many previous situations in which she felt mistreated by ‘the system’, therefore she no longer wanted to work for that ‘system’ and only wanted to perform undeclared work. Furthermore, patients with BPD described that they often felt misunderstood outside the mental health care system. Therefore, they would rather start exploring ways to (re-)start employment during the course of their treatment. Furthermore, this exploration preferably took place with one designated person to discuss potential difficulties and support in gaining and maintaining employment and to whom they could potentially return.

All groups expressed that collaboration between mental health services and vocational rehabilitation should be improved to enhance (support to) employment in patients with BPD. Most patients described that in previous working experiences a working environment in which they felt comfortable and accepted was the most important aspect. Some patients described how work distracted them from symptoms of BPD such as mood swings and negative thoughts. Two patients with BPD referred to the need of feeling comfortable within their working environment and of being personally responsible for clearly defined tasks, as described by participant 8: *“[The best working conditions in the past constituted ] feeling secure with the colleagues around me*, *I suppose*, *and having my own little enterprise” [in which clearly defined personal tasks were performed]*. Both MHPs and IPs described a similar working climate necessary, in which a certain amount of freedom with clearly defined tasks was key. Concurrently, MHPs and IPs endorsed the need of a match between work context and the individual with BPD (with personal needs and characteristics).

## Discussion

In the present study, barriers and facilitators to employment in BPD were studied by interviewing patients, mental health practitioners, and insurance physicians. We found that the identified barriers and facilitators related to three overall themes: characteristics of BPD, stigma and support to employment. Generally barriers and facilitators corresponded to identical features, revealing an interactive process within each theme. The suggested facilitators provided key elements of targeting the identified barriers. Overall, more barriers than facilitators were mentioned by all groups, especially when BPD symptoms were not treated. Also identified barriers were mostly related to maintaining employment and less to gaining employment, which seems different than for other severe mental disorders.

### Characteristics of BPD

According to all participants barriers mainly related to symptoms of BPD. This finding is consistent with literature proposing a link between the core symptoms of BPD (mood swings and problems in interpersonal relationships and self-image) and multiple areas of impaired functioning [1,6,47]. Although patients with BPD stated to have the ability and wish to work with others, they simultaneously felt misunderstood and reported low self-image and difficulties in posing personal boundaries. This corresponds to previous findings showing that although patients with BPD accurately sensed and connected to the emotions of others, their understanding and contextualizing of emotions and thoughts of others was impaired compared to healthy controls [48,49]. Furthermore, patients with BPD explained how multiple problems from different domains of life further aggravated their sense of loss of overview, also affecting their job. Previous studies showing a chronic state of heightened affect in patients with BPD [50] could explain this vicious circle of additional problems typical in BPD. We additionally noted that, in contrast to other severe mental disorders, where a lack of motivation or work experience mainly hindered gaining employment [24,32], BPD patients in our study experienced difficulty in maintaining employment and adequately regulating emotions at work.

The participants in the present study explained that treatment is needed to diminish symptoms and thereby increase functioning. In turn, being employed was found to naturally diminish BPD symptoms [7] and increase self-reflection [13]. According to MHPs and IPs, externalization and overestimation of patients with BPD resulted in pursuing unsuitable jobs. Due to a difficulty in regulating emotions, patients with BPD were often overwhelmed by their emotions and consequently had lessened understanding of their behavior resulting from these emotions. This low self-awareness, self-reflection, and self-directedness in BPD were previously described as being the cause of externalization and overestimation in BPD [51,52]. Furthermore, patients with BPD are more likely to report on problems as caused by others [36]. However, Horn and colleagues [53] argue that externalization in patients with BPD should be used to move away from “hopelessness” and the “personality disorder” label. Acknowledging externalization and simultaneously challenging thoughts and feelings of rejection can be used to find ways for patient and practitioner to break out of a vicious cycle of detrimental interplay. To some extent this was also found in the present study, as IPs observed a different attitude in patients with BPD when following patients’ job wishes. However, this did not always lead to successful placements.

### Stigma

In line with previous literature, patients with BPD felt great antipathy towards the “borderline” label although they appreciated receiving support and therapy based on their diagnosis [53,54]. In addition, MHPs and IPs realized being biased themselves about the capabilities of patients with BPD to work. Previous studies showed that negative attitudes of professionals towards the capacity to gain employment impeded gaining and maintaining employment in patients with mental illnesses [55,56]. This may also hold for patients with BPD.

Furthermore, Bungert and colleagues [57] previously suggested that the negative attitudes of professionals could increase feelings of rejection and abandonment in patients with BPD. Simultaneously, both MHPs and IPs realized being at risk of inducing anticipated stigma in patients with BPD by having little hope for improvement in functioning. This anticipated stigma from professionals was previously argued to impede gaining employment [45]. Both patients with BPD and MHPs suggested that BPD should be renamed ‘emotion regulation disorder’ in an attempt to facilitate disclosure of the diagnosis to employers and coworkers. Simultaneously, disclosure could serve as a means to communicate needs and adjust working conditions accordingly, ultimately increasing sustainable employment and targeting stigma [58–60]. Among patients however, fear of stigma and discrimination was an important reason for non-disclosure. Goldberg and colleagues [61] demonstrated that the choice of disclosure was related to the individuals’ phase of recovery, suggesting that those ‘further’ in the recovery process were better able to manage their symptoms and skills. This was confirmed by the patients from the present that already received treatment for some time. Furthermore, professionals explained that an increase of self-reflection (through treatment) was needed to increase sustainable employment. In addition to previous studies on barriers and facilitators to employment in mental health disorders, the present study suggested useful strategies for practice, such as developing a manual to manage disclosure and promoting the positive features of BPD in the public domain to target stigma.

### Support to employment

An important facilitator identified in our study was that most patients with BPD wanted to be employed (in the future) and expressed hope of achieving this goal. This is essential since the motivation to be employed is found to be a predictor of sustainable employment in individuals with mental illness (next to job match, support and self-confidence) [32,62]. IPs acknowledged the importance of motivation for employment, yet generally perceived the desired job of patients with BPD as unsuitable. However, matching job wishes and following patient preferences are key elements of supported employment and important facilitators for sustainable employment [32,62,63].

The need to increase collaboration between mental health and vocational rehabilitation services was endorsed by all participants. Patients with BPD found support to employment strategies fragmented and not fitting their needs. Previous studies addressed this lack of support and insufficient collaboration between mental health services and SSA in individuals with diverse mental health problems [25–27,64]. In line with our findings, these studies showed that a lack of collaboration between services together with having problems in different domains of life, next to mental health problems, affected return to employment. They stated that more support is needed in addressing these problems in order to sustainably return to work. In addition to this literature the present study demonstrates that the sometimes diverging perspectives of patients and professionals requires a better understanding of BPD to better match adequate support.

Integrating vocational rehabilitation services within mental health care following patient preferences and providing long-term support are key principles of the evidence-based supported employment method IPS [65]. This method, originally developed for patients with severe mental illness, centers on the principle of direct employment without preceding training, while focusing on participants’ preferences and the assumption that everyone with a wish to gain employment should have the opportunity to find regular paid employment [66–68]. Given the identified barriers in this study, IPS thus seems to be a particularly suitable method of supported employment in BPD. Currently in the Netherlands however, although all individuals regardless of type of mental disorder are eligible for vocational rehabilitation, IPS is only available for patients in FACT care. This means that patients in specialized treatment programs for BPD currently have no access to IPS. Importantly, IPS has recently also been shown to be effective in other populations than in patients with severe mental illness, such as patients with post-traumatic stress disorder, common mental disorders and substance use disorders [69]. Bond and colleagues (2019) suggest that modifications in the IPS program might be needed in these patient groups as they are often heterogeneous and in need of an individualized approach, which is in line with the pragmatic principles of IPS not being specific to any impairment or condition. This may also hold for IPS in patients with BPD as they often have heterogeneous symptoms, significant comorbidity and outspoken wishes for employment, which are, according to professionals, not always easy to match.

### Strengths and limitations

To the best of our knowledge this is the first study qualitatively examining barriers and facilitators to employment in BPD among patients, MHPs and IPs. Strengths of the current study include: 1) the triangulation of perspectives from patients, MHPs and IPs as assessed with both in-depth individual interviews and focus group interviews, 2) the comparison between different perspectives from two fields of practice, and 3) the broad sample of patients with BPD constituting those with diverse backgrounds in age, work history and treatment history. The study, however, also has limitations. First, snowballing was used to include eligible participants, which might have led to selection bias. Second, it is conceivable that patients with a less favorable attitude towards employment were not interested in participating, leading to an overestimation of the perceived importance of employment in BPD (especially since we had little information about non-responders). Third, patients from the present study represent a selective group of BPD patients that are in specialized treatment programs for BPD. A significant portion of BPD patients are not in treatment [6,70]. Clearly, our results do not generalize to all individuals with BPD. Fourth, we did not study the interplay between patients and both professionals groups, which would have extended our findings. However, from this first explorative study we found that perspectives diverged. Therefore, future research could study the interplay between patient and professional (dyads) in a multiple case study design. Fifth, the perspectives of employers were not explored which causes the results to be relatively less applicable to the pathway of maintaining employment. Sixth and finally, most patients with BPD in the present study also had other mental disorders, that by themselves have been shown to impair employment. Likewise, comorbidity of BPD with affective disorders was found to increase occupational impairment [18]. Although during the interviews we consistently aimed to distinguish the barriers and facilitators originating from BPD from those originating from possible comorbid disorders, we cannot completely disentangle them in this study. In addition, severity of BPD has been argued to be a determinant of impairment in occupational functioning [71,72].

### Conclusions and implications for practice and further research

The identified barriers and facilitators guide future research into employment in BPD and suggest that support to employment in individuals with BPD can be enhanced. The present findings clearly suggest that diminishing symptoms, examining stigma and increasing support to employment could serve as starting points for future research. Most identified facilitators correspond to important elements of evidence-based support programs to employment, such as IPS. These programs have a patient-centered approach and integrate mental health and vocational services [63]. Studying the effectiveness of IPS, which so far has been primarily examined in the context of severe mental illness in general [73–76], may be a promising first step. In studying support to employment for BPD, key elements should be 1) acknowledging a potential divergent perspective in professionals and patients about suitability of pursued employment, and 2) examining the role of stigma and disclosure in the pathway of gaining and maintaining employment for patients with BPD.

## Supporting information

S1 File Topic list. (PDF)

S1 Table Characteristics of patients with BPD. (DOCX)

## References

[1] American Psychiatric Association. (2013) Diagnostic and statistical manual of mental disorders (5th ed.) Washington, DC: American Journal of Psychiatry 10.1176/appi.books.9780890425596.744053

[2] Samuels J, Eaton WW, Bienvenu OJ, Brown C, Costa PT Jr., Nestadt G. Prevalence and correlates of personality disorders in a community sample. Br J Psychiatry. 2002;180: 536–542. 10.1192/bjp.180.6.536 12042233

[3] Coid J, Yang MIN, Tyrer P, Roberts A, Ullrich S. Prevalence and correlates of personality disorder in Great Britain. Br J Psychiatry. 2006;188: 423–431. 10.1192/bjp.188.5.423 16648528

[4] Lenzenweger MF, Lane MC, Loranger AW, Kessler RC. DSM-IV Personality Disorders in the National Comorbidity Survey Replication. Biol Psychiatry. 2007;62: 553–564. 10.1016/j.biopsych.2006.09.019 17217923 PMC2044500

[5] Paris J. Estimating the Prevalence of Personality Disorders in the Community. J Pers Disord. 2010;24 10.1521/pedi.2010.24.4.405 20695802

[6] Leichsenring F., Leibing E., Kruse J., New A. S., Leweke F. Borderline Personality Disorder. Lancet. Elsevier Ltd; 2011;377: 74–84. 10.1016/S0140-6736(10)61422-5 21195251

[7] Cruitt PJ, Boudreaux MJ, Jackson JJ, Oltmanns TF. Borderline Personality Pathology and Physical Health: The Role of Employment. Personal Disord Theory, Res Treat. 2016; 9 10.1037/per0000211 PMC5311027 27657166

[8] Jovev M, Jackson HJ. The relationship of borderline personality disorder, life events and functioning in an Australian psychiatric sample. J Pers Disord. 2006;20: 205–217. 10.1521/pedi.2006.20.3.205 16776551

[9] Skodol AE, Gunderson JG, McGlashan TH, Dyck IR, Stout RL, Bender DS, et al Functional impairment in patients with schizotypal, borderline, avoidant, or obsessive-compulsive personality disorder. Am J Psychiatry. 2002;159: 276–83. 10.1176/appi.ajp.159.2.276 11823271

[10] Hengartner MP, Müller M, Rodgers S, Rössler W, Ajdacic-Gross V. Occupational functioning and work impairment in association with personality disorder trait-scores. Soc Psychiatry Psychiatr Epidemiol. 2014;49: 327–335. 10.1007/s00127-013-0739-2 23835577

[11] McGurk SR, Mueser KT, Mischel R, Adams R, Harvey PD, McClure MM, et al Vocational functioning in schizotypal and paranoid personality disorders. Psychiatry Res. Elsevier; 2013;210: 498–504. 10.1016/j.psychres.2013.06.019 23932840

[12] Zimmerman M, Martinez JH, Young D, Chelminski I, Dalrymple K. Sustained unemployment in psychiatric outpatients with bipolar depression compared to major depressive disorder with comorbid borderline personality disorder. Bipolar Disord. 2012;14: 856–862. 10.1111/bdi.12014 23057759

[13] Katsakou C, Marougka S, Barnicot K, Savill M, White H, Lockwood K, et al Recovery in borderline personality disorder (BPD): A qualitative study of service users’ perspectives. PLoS One. 2012;7: 1–8. 10.1371/journal.pone.0036517 22615776 PMC3355153

[14] Alvarez-Jimenez M, Gleeson JF, Henry LP, Harrigan SM, Harris MG, Killackey E, et al Road to full recovery: longitudinal relationship between symptomatic remission and psychosocial recovery in first-episode psychosis over 7.5 years. Psychol Med. 2012;42: 595–606. 10.1017/S0033291711001504 21854682

[15] Amundsen Ostby K, Czajkowski N, Knudsen GP, Ystrom E, Gjerde LC, Kendler KS, et al Personality disorders are important risk factors for disability pensioning. Soc Psychiatry Psychiatr Epidemiol. 2014;49: 2003–2011. 10.1007/s00127-014-0878-0 24791656 PMC4218874

[16] Gunderson JG, Stour RL, McGlashan TH, Shea MT, Morey LC, Grilo CM, et al Ten-Year Course of Borderline Personality Disorder. Arch Gen Psychiatry. 2011;68: 827 10.1001/archgenpsychiatry.2011.37 21464343 PMC3158489

[17] Knudsen AK, Skogen JC, Harvey SB, Stewart R, Hotopf M, Moran P. Personality disorders, common mental disorders and receipt of disability benefits: evidence from the British National Survey of Psychiatric Morbidity. Psychol Med. 2012;42: 2631–40. 10.1017/S0033291712000906 22565011

[18] Korkeila J, Oksanen T, Virtanen M, Salo P, Nabi H, Pentti J, et al Early retirement from work among employees with a diagnosis of personality disorder compared to anxiety and depressive disorders. Eur Psychiatry. 2011;26: 18–22. 10.1016/j.eurpsy.2009.12.022 20541917

[19] Wagner T, Fydrich T, Stiglmayr C, Marschall P, Salize HJ, Renneberg B, et al Societal cost-of-illness in patients with borderline personality disorder one year before, during and after dialectical behavior therapy in routine outpatient care. Behav Res Ther. Elsevier Ltd; 2014;61: 12–22. 10.1016/j.brat.2014.07.004 25113523

[20] Zanarini MC, Jacoby RJ, Frankenburg FR, Reich DB, Fitzmaurice G. The 10-year course of social security disability income reported by patients with borderline personality disorder and axis II comparison subjects. J Pers Disord. 2009;23: 346–356. 10.1521/pedi.2009.23.4.346 19663655 PMC3222934

[21] Hoedeman R. OECD. Sick on the job? Myths and realities about mental health and work. OECD. 2012 10.1007/s12498-012-0114-3

[22] Burns T, Catty J, White S, Becker T, Koletsi M, Fioritti A, et al The impact of supported employment and working on clinical and social functioning: Results of an international study of individual placement and support. Schizophr Bull. 2008;35: 949–958. 10.1093/schbul/sbn024 18403375 PMC2728809

[23] Kinn LG, Holgersen H, Aas RW, Davidson L. “Balancing on Skates on the Icy Surface of Work”: A metasynthesis of work participation for persons with psychiatric disabilities. J Occup Rehabil. 2014;24: 125–138. 10.1007/s10926-013-9445-x 23653177

[24] Koletsi M, Niersman A, van Busschbach JT, Catty J, Becker T, Burns T, et al Working with mental health problems: Clients’ experiences of IPS, vocational rehabilitation and employment. Soc Psychiatry Psychiatr Epidemiol. 2009;44: 961–970. 10.1007/s00127-009-0017-5 19280083

[25] Audhoe SS, Nieuwenhuijsen K, Hoving JL, Sluiter JK, Frings-Dresen MHW. Perspectives of unemployed workers with mental health problems: barriers to and solutions for return to work. Disabil Rehabil; 2016; 1–7. 10.1080/09638288.2016.1242170 27756177

[26] Lammerts L, Schaafsma FG, van Mechelen W, Anema JR. Execution of a participatory supportive return to work program within the Dutch social security sector: a qualitative evaluation of stakeholders’ perceptions. BMC Public Health; 2016;16:323 10.1186/s12889-016-2997-x 27074885 PMC4831193

[27] Vukadin M, Schaafsma FG, Westerman MJ, Michon HWC, Anema JR. Experiences with the implementation of Individual Placement and Support for people with severe mental illness: A qualitative study among stakeholders. BMC Psychiatry; 2018;18: 145 10.1186/s12888-018-1729-4 29793455 PMC5968490

[28] Noel VA, Oulvey E, Drake RE, Bond GR, Elizabeth A, Deatley B. A Preliminary Evaluation of Individual Placement and Support for Youth with Developmental and Psychiatric Disabilities Developmental and Psychiatric Disabilities. J of Voc Rehab. 2018; 48;2 10.3233/JVR-180934

[29] Brouwers EPM, Mathijssen J, Van Bortel T, Knifton L, Wahlbeck K, Van Audenhove C, et al Discrimination in the workplace, reported by people with major depressive disorder: a cross-sectional study in 35 countries. BMJ Open. 2016;6: e009961 10.1136/bmjopen-2015-009961 26908523 PMC4769412

[30] Alverson H, Carpenter E, Drake RE. An ethnographic study of job seeking among people with severe mental illness. Psychiatr Rehabil J. 2006;30: 15–22. 10.2975/30.2006.15.22 16881241

[31] Becker D, Whitley R, Bailey EL, Drake RE. Long-term Employment Trajectories Among Participants with Severe Mental Illness in Supported Employment. Psychiatric Services; 2007;58:922–928. 10.1176/ps.2007.58.7.922 17602007

[32] Kukla M, McGuire AB, Salyers MP. Barriers and Facilitators Related to Work Success for Veterans in Supported Employment: A Nationwide Provider Survey. Psychiatr Serv. 2015; 1–6. 10.1176/appi.ps.660102 26695496

[33] Catty J, Lissouba P, White S, Becker T, Drake RE, Fioritti A, et al Predictors of employment for people with severe mental illness: Results of an international six-centre randomised controlled trial. Br J Psychiatry. 2008;192: 224–231. 10.1192/bjp.bp.107.041475 18310585

[34] Sansone RA, Sansone LA. The interface: Employment in Borderline Personality Disorder. Innov Clin Neurosci. 2012;9: 25–29. PMC3472897 23074700

[35] Sio IT, Chanen AM, Killackey EJ, Gleeson J. The relationship between impulsivity and vocational outcome in outpatient youth with borderline personality features. Early Interv Psychiatry. 2011;5: 249–253. 10.1111/j.1751-7893.2011.00271.x 21521492

[36] Catthoor K, Feenstra DJ, Hutsebaut J, Schrijvers D, Sabbe B. Adolescents with personality disorders suffer from severe psychiatric stigma: evidence from a sample of 131 patients. Adolesc Health Med Ther. 2015;6: 81–9. 10.2147/AHMT.S76916 25999774 PMC4427063

[37] Sheehan L, Nieweglowski K, Corrigan P. The Stigma of Personality Disorders. Curr Psychiatry Rep. 2016;18: 1–7. 10.1007/s11920-015-0646-1 26780206

[38] Aviram RB, Brodsky BS, Stanley B. Borderline Personality Disorder, Stigma, and Treatment Implications. Harv Rev Psychiatry. 2006;14: 249–256. 10.1080/10673220600975121 16990170

[39] Bodner E, Cohen-Fridel S, Mashiah M, Segal M, Grinshpoon A, Fischel T, et al The attitudes of psychiatric hospital staff toward hospitalization and treatment of patients with borderline personality disorder. BMC Psychiatry. 2015;15: 2 10.1186/s12888-014-0380-y 25609479 PMC4307152

[40] Dickens GL, Hallett N, Lamont E. Interventions to improve mental health nurses' skills attitudes, and knowledge related to people with a diagnosis of borderline personality disorder: systematic review. Int J of Nursing Studies. 2016;114–127. 10.1016/j.ijnurstu.2015.10.019 26747180

[41] Lugtenberg M, van Beurden KM, Brouwers EPM, Terluin B, van Weeghel J, van der Klink JJL, et al Occupational physicians’ perceived barriers and suggested solutions to improve adherence to a guideline on mental health problems: analysis of a peer group training. BMC Health Serv Res. BMC Health Services Research; 2016;16: 271–282. 10.1186/s12913-016-1530-3 27423463 PMC4947285

[42] van Rooijen S, van Weeghel J. Jaarboek Psychiatrische Rehabilitatie 2011–2012. 1st ed Amsterdam: SWP Uitgeverij BV; 2010.

[43] Drukker M, Maarschalkerweerd M, Bak M, Driessen G, à Campo J, de Bie A, et al A real-life observational study of the effectiveness of FACT in a Dutch mental health region. BMC Psychiatry. 2008;8: 93 10.1186/1471-244X-8-93 19055813 PMC2629765

[44] Green J, Thorogood N. Qualitative Methods for Health Research. Introducing qualitative methods. 2014 10.1177/1049732305285708

[45] Brouwers E. Stigma op psychische problemen is een barrière voor arbeidsparticipatie. Tijdschr voor Bedrijfs- en Verzek. 2016;24: 155–157.

[46] Sevak P, Khan S. Psychiatric Versus Physical Disabilities: A Comparison of Barriers and Facilitators to Employment. Psychiatr Rehabil J. 2016 10.1037/prj0000236 27786522

[47] Schuppert HM, Giesen-Bloo J, Van Gemert TG, Wiersema HM, Minderaa RB, Emmelkamp PMG, et al Effectiveness of an emotion regulation group training for adolescents—A randomized controlled pilot study. Clin Psychol Psychother. 2009;16: 467–478. 10.1002/cpp.637 19630069

[48] Harari H, Shamay-Tsoory SG, Ravid M, Levkovitz Y. Double dissociation between cognitive and affective empathy in borderline personality disorder. Psychiatry Res. Elsevier Ireland Ltd; 2010;175: 277–279. 10.1016/j.psychres.2009.03.002 20045198

[49] Mier D, Lis S, Esslinger C, Sauer C, Hagenhoff M, Ulferts J, et al Neuronal correlates of social cognition in borderline personality disorder. Soc Cogn Affect Neurosci. 2013;8: 531–537. 10.1093/scan/nss028 22362841 PMC3682436

[50] Lazarus SA, Cheavens JS, Festa F, Zachary Rosenthal M. Interpersonal functioning in borderline personality disorder: A systematic review of behavioral and laboratory-based assessments. Clin Psychol Rev. Elsevier Ltd; 2014;34: 193–205. 10.1016/j.cpr.2014.01.007 24534643

[51] Bateman AW, Gunderson J, Mulder R. Treatment of personality disorder. Lancet. Elsevier Ltd; 2015;385: 735–743. 10.1016/S0140-6736(14)61394-5 25706219

[52] Tyrer P, Reed GM, Crawford MJ. Classification, assessment, prevalence, and effect of personality disorder. Lancet. 2015;385: 717–726. 10.1016/S0140-6736(14)61995-4 25706217

[53] Horn N, Johnstone L, Brooke S. Some service user perspectives on the diagnosis of Borderline Personality Disorder. J Ment Heal. 2007;16: 255–269. 10.1080/09638230601056371

[54] Nehls N. Borderline personality disorder: The voice of patients. Res Nurs Heal. 1999;22: 285–293. 10.1002/(SICI)1098-240X(199908)22:4<285::AID-NUR3>3.0.CO;2-R 10435546

[55] Pogoda TK, Cramer IE, Rosenheck RA, Resnick SG. Qualitative analysis of barriers to implementation of supported employment in the Department of Veterans Affairs. Psychiatr Serv. 2011;62: 1289–1295. 10.1176/ps.62.11.pss6211_1289 22211207

[56] Swanson S, Burson K, Harper J, Johnson B, Litvak J, McDowell M, et al Implementation issues for IPS supported employment: Stakeholders share their strategies. Am J Psychiatr Rehabil. 2011;14: 165–180. 10.1080/15487768.2011.598099

[57] Bungert M, Liebke L, Thome J, Haeussler K, Bohus M, Lis S. Rejection sensitivity and symptom severity in patients with borderline personality disorder: effects of childhood maltreatment and self-esteem. Borderline Personal Disord Emot dysregulation. Borderline Personality Disorder and Emotion Dysregulation; 2015;2: 13 10.1186/s40479-015-0034-9 26401307 PMC4579499

[58] Corrigan P. On the Self-Stigma of Mental Illness: Stages, Disclosure, and Strategies for Change. Can J Psychiatry. 2012;57: 464–469. 10.1177/070674371205700804 22854028 PMC3610943

[59] Corrigan PW, Rüsch N, Scior K. Adapting Disclosure Programs to Reduce the Stigma of Mental Illness. Psychiatr Serv. 2018;69: 826–828. 10.1176/appi.ps.201700478 29606076

[60] Allott KA, Turner LR, Chinnery GL, Killackey EJ, Nuechterlein KH. Managing disclosure following recent-onset psychosis: Utilizing the Individual Placement and Support model. Early Interv Psychiatry. 2013;7: 338–344. 10.1111/eip.12030 23343040

[61] Goldberg SG, Killeen MB, O’Day B. The disclosure conundrum: How people with psychiatric disabilities navigate employment. Psychol Public Policy, Law. 2005;11: 463–500. 10.1037/1076-8971.11.3.463

[62] Reddy LF, Llerena K, Kern RS. Predictors of employment in schizophrenia: The importance of intrinsic and extrinsic motivation. Schizophr Res. Elsevier B.V.; 2016;176: 462–466. 10.1016/j.schres.2016.08.006 27567733

[63] Becker DR, Swanson SJ, Reese SL, Bond GR, Bethany M. Supported Employment Review Manual. 3rd ed Dartmouth Psychiatric Research Center; 2015.

[64] Noel VA, Oulvey E, Drake RE, Bond GR, Carpenter-Song EA, Deatley B. A preliminary evaluation of individual placement and support for youth with developmental and psychiatric disabilities. J Vocat Rehabil. 2018;48: 249–255. 10.3233/JVR-180934

[65] Drake RE, Bond GR. IPS Support Employment: a 20-year Update. Am J Psychiatr Rehabil. 2011;14: 155–164. 10.1080/15487768.2011.598090

[66] Becker D, Drake R, Bond G. Benchmark Outcomes in Supported Employment. Am J Psychiatr Rehabil. 2011;14: 230–236. 10.1080/15487768.2011.598083

[67] Bond GR, Kim SJ, Becker DR, Swanson SJ, Drake RE, Krzos IM, et al A Controlled Trial of Supported Employment for People With Severe Mental Illness and Justice Involvement. Psychiatr Serv. 2015;66: 1027–1034. 10.1176/appi.ps.201400510 26030319

[68] Burns T, Catty J, Becker T, Drake RE, Fioritti A, Knapp M, et al The effectiveness of supported employment for people with severe mental illness: a randomised controlled trial. Lancet. 2007;370: 1146–1152. 10.1016/S0140-6736(07)61516-5 17905167

[69] Bond GR, Drake RE, Pogue JA. Expanding Individual Placement and Support to Populations With Conditions and Disorders Other Than Serious Mental Illness. Psychiatr Serv. 2019 10.1176/appi.ps.201800464 30813865

[70] Coid J., Yang M., Beppington P., Moran P., Brugha T., Jenkins R., Farrell M., Singleton N. Borderline personality disorder: health service use and social functioning among a national household population. Psychol Med. 2009;39: 1721–1731. 10.1017/S0033291708004911 19250579

[71] Tyrer P. Personality disorder and public mental health. Clin Med (Northfield Il). 2008;8: 423–427. 10.7861/clinmedicine.8-4-423 18724613 PMC4952939

[72] Yang M, Coid J, Tyrer P. Personality pathology recorded by severity: National survey. Br J Psychiatry. 2010;197: 193–199. 10.1192/bjp.bp.110.078956 20807963

[73] Sveinsdottir V, Løvvik C, Fyhn T, Monstad K, Ludvigsen K, Øverland S, et al Protocol for the effect evaluation of Individual Placement and Support (IPS): a randomized controlled multicenter trial of IPS versus treatment as usual for patients with moderate to severe mental illness in Norway. BMC Psychiatry. 2014;14: 307 10.1186/s12888-014-0307-7 25403470 PMC4244061

[74] Burns T, Catty J, Becker T, Drake RE, Fioritti A, Knapp M, et al The effectiveness of supported employment for people with severe mental illness: a randomised controlled trial. Lancet (London, England). 2007;370: 1146–52. 10.1016/S0140-6736(07)61516-5 17905167

[75] Tsang HWH. Supported employment versus traditional vocational rehabilitation for individuals with severe mental illness: a three-year study. Hong Kong Med J. 2011;17 Suppl 2: 13. 21368328

[76] Michon H, van Busschbach JT, Stant a D, van Vugt MD, van Weeghel J, Kroon H. Effectiveness of individual placement and support for people with severe mental illness in The Netherlands: a 30-month randomized controlled trial. Psychiatr Rehabil J. 2014;37: 129–36. 10.1037/prj0000061 24912062

