# Supplementary material for: Barriers and facilitators to employment in borderline personality disorder: A qualitative study among patients, mental health practitioners and insurance physicians
Source: PLoS One. 2019 Jul 23;14(7):e0220233. doi: 10.1371/journal.pone.0220233 (PMC6650068; doi:10.1371/journal.pone.0220233)
Supplement: S1 File — (PDF) [file pone.0220233.s001.pdf]

S1 File. Topic list

Sociodemographic information:
- Age
- Level of education
- Living situation

Employment:
- Current situation?
- Employment history
- Work experience last 5 years
- (Social) benefits?
- Vocational rehabilitation trajectories? (current & past)

General experience with employment/vocational rehabilitation
- Positive & negative experience (decision latitude, psychological job demands, job security, and social support) in relation to previous work experience if possible
- Support from Social Security Administration/municipality?

Self-awareness
- Expectations of being employed?
- Motivation to be employed?
- Advantages & disadvantages of being employed?

Symptoms
- Facilitating/ impeding

Support
- Experienced support?
- Missed support?

Stigma
- Anticipated stigma
- Discrimination

Disclosure
