# Supplementary material for: Barriers and facilitators to employment in borderline personality disorder: A qualitative study among patients, mental health practitioners and insurance physicians
Source: PLoS One. 2019 Jul 23;14(7):e0220233. doi: 10.1371/journal.pone.0220233 (PMC6650068; doi:10.1371/journal.pone.0220233)
Supplement: S1 Table — (DOCX) [file pone.0220233.s002.docx]

**S1 Table. Characteristics of patients with BPD**

| Participant (n=15) | Gender | Age | Employment situation and income | Living situation | Borderline personality disorder diagnosed (DSM-IV) | Comorbid psychiatric disorder |
| --- | --- | --- | --- | --- | --- | --- |
| 1 | Female | 34 | Employed, housekeeping (approximately 10h per week)  Previously fulltime employed  Additional social welfare benefits income (municipality) | Single, living with son | October 2015 | Post-Traumatic Stress Disorder |
| 2 | Female | 30 | Employed, civil servant (18h per week)  Previously fulltime employed | Single, living with mother | June 2016 | Paranoid personality disorder |
| 3 | Female | 46 | Employed, hospitality sector (10h per week)  Previously employed in retail (32h per week)  Additional sickness benefits income (SSA) | Recently divorced, room with shared facilities | March 2016 | Generalized anxiety disorder |
| 4 | Female | 23 | Unemployed since approximately 4 months  Previously employed in housekeeping (non-fixed hours)  Sickness benefits income (SSA) | Single, no permanent residence | December 2016 | Depressive disorder,  Substance use disorder, |
| 5 | Female | 49 | Unemployed  Recently quitted voluntary job in child care  Sickness benefits income from the disability Act (WAO – incapacitated for work) (SSA) | Single | November 2004 | Eating disorder |
| 6 | Female | 43 | Unemployed but in voluntary job hospitality sector  (1 day per week)  Social welfare benefits income (Municipality) | Single | July 2010 | Dissociative disorder, |
| 7 | Female | 26 | Unemployed  Previously employed in child care (fulltime)  Social welfare benefits income (Municipality) | Single, temporary housing | April 2015 | Depressive disorder |
| 8 | Female | 58 | Employed, administrative officer (24/25h per week)  Previous 36 years fulltime  Since recently, partially in sickness benefits (SSA) | Single | September 2012 | Depressive disorder,  Avoidant personality disorder |
| 9 | Female | 33 | Intern at government institution (fulltime)  Previous 5 years in sickness benefits (SSA) | Living together | January 2012 | None |
| 10 | Male | 36 | Unemployed  Previous 13 years employed in retail (fulltime)  Sickness benefits income (SSA) | Single | May 2017 | None |
| 11 | Female | 48 | Unemployed  Previously employed in retail (32h per week)  Sickness benefits income (SSA) | Single, living with daughter | February 2011 | Attention Deficit Hyperactivity Disorder (AD/HD),  Substance use disorder |
| 12 | Female | 34 | Unemployed  Previously employed as administrative officer (fulltime)  Sickness benefits income (SSA) | Single | June 2015 | AD/HD |
| 13 | Female | 36 | Unemployed but in voluntary job in multimedia (without fixed hours)  Social welfare benefits income (Municipality) | Living apart together (LAT) | April 2016 | Depressive disorder,  Generalized anxiety disorder,  AD/HD |
| 14 | Female | 40 | Unemployed  Sickness benefits income from the disability Act (WAO - incapacitated for work) (SSA) | Single living with children | February 2016 | Bipolar disorder,  PTSD |
| 15 | Female | 50 | Unemployed but side earnings from unregistered jobs (without fixed hours)  Social welfare benefits income (Municipality) | Single living with son | January 2015 | Substance use disorder, |

SSA: Social Security Administration

PTSD: Post-Traumatic Stress Disorder

AD/HD: Attention Deficit Hyperactivity Disorder
